# Effects of Acute Exposure to Moderate Altitude on Vascular Function, Metabolism and Systemic Inflammation

**DOI:** 10.1371/journal.pone.0070081

**Published:** 2013-08-01

**Authors:** Anne-Christin Stöwhas, Tsogyal D. Latshang, Christian M. Lo Cascio, Sina Lautwein, Katrin Stadelmann, Noemi Tesler, Lisa Ayers, Kaspar Berneis, Philipp A. Gerber, Reto Huber, Peter Achermann, Konrad E. Bloch, Malcolm Kohler

**Affiliations:** 1 Pulmonary Division, University Hospital Zurich, Zurich, Switzerland; 2 Institute of Pharmacology and Toxicology, University of Zurich, Zurich, Switzerland; 3 Child Development Centre, University Children's Hospital Zurich, Zurich, Switzerland; 4 Department of Clinical Immunology, Churchill Hospital, Oxford, United Kingdom; 5 Department of Endocrinology, Diabetes and Nutrition, University Hospital Zurich, Zurich, Switzerland; 6 Zurich Center for Integrative Human Physiology (ZIHP), University of Zurich, Zurich, Switzerland; Tulane School of Public Health and Tropical Medicine, United States of America

## Abstract

**Background:**

Travel to mountain areas is popular. However, the effects of acute exposure to moderate altitude on the cardiovascular system and metabolism are largely unknown.

**Objectives:**

To investigate the effects of acute exposure to moderate altitude on vascular function, metabolism and systemic inflammation.

**Methods:**

In 51 healthy male subjects with a mean (SD) age of 26.9 (9.3) years, oxygen saturation, blood pressure, heart rate, arterial stiffness, lipid profiles, low density lipoprotein (LDL) particle size, insulin resistance (HOMA-index), highly-sensitive C-reactive protein and pro-inflammatory cytokines were measured at 490 m (Zurich) and during two days at 2590 m, (Davos Jakobshorn, Switzerland) in randomized order. The largest differences in outcomes between the two altitudes are reported.

**Results:**

Mean (SD) oxygen saturation was significantly lower at 2590 m, 91.0 (2.0)%, compared to 490 m, 96.0 (1.0)%, p<0.001. Mean blood pressure (mean difference +4.8 mmHg, p<0.001) and heart rate (mean difference +3.3 bpm, p<0.001) were significantly higher at 2590 m, compared to 490 m, but this was not associated with increased arterial stiffness. At 2590 m, lipid profiles improved (median difference triglycerides −0.14 mmol/l, p = 0.012, HDL +0.08 mmol/l, p<0.001, total cholesterol/HDL-ratio −0.25, p = 0.001), LDL particle size increased (median difference +0.45 nm, p = 0.048) and hsCRP decreased (median difference −0.18 mg/l, p = 0.024) compared to 490 m. No significant change in pro-inflammatory cytokines or insulin resistance was observed upon ascent to 2590 m.

**Conclusions:**

Short-term stay at moderate altitude is associated with increased blood pressure and heart rate likely due to augmented sympathetic activity. Exposure to moderate altitude improves the lipid profile and systemic inflammation, but seems to have no significant effect on glucose metabolism.

**Trial Registration:**

ClinicalTrials.gov NCT01130948

## Introduction

Travel to mountain areas for leisure activities or a professional reason has become increasingly popular for lowland residents. It has been estimated that several million people in the US and Europe travel to moderate altitude each year, many of which have known or unknown arterial hypertension, diabetes mellitus, disturbances in lipid metabolism and cardiovascular disease. Understanding the interactions between hypoxia, measures of cardiovascular function and metabolism is an important requirement for counselling persons travelling to altitude. However, the acute effects of moderate altitude exposure on measures of cardiovascular function and metabolism are still incompletely defined. This is because very few studies, most of which included a small number of subjects and differed regarding the duration of altitude exposure, looked at the effects of moderate altitude on measures of cardiovascular function, glucose and lipid metabolism. In addition, the studied populations differed considerably, with some studies including healthy men and women and others including patients with several comorbidities, thus resulting in contradictory findings do not allow to draw final conclusions [1]–[6]. However, hypobaric hypoxia has previously been shown to be associated with increased sympathetic activity which in turn may have a substantial effect not only on blood pressure but also on glucose and lipid metabolism [7]–[9].

We have addressed this uncertainty by performing a randomized, controlled, cross-over trial investigating the effects of acute exposure to moderate altitude on measures of cardiovascular function, glucose and lipid metabolism as well as on measures of systemic inflammation in healthy male adults. We hypothesized that acute exposure to moderate altitude leads to hypoxemia associated with worsening of cardiovascular function, glucose and lipid metabolism as well as with an increase in systemic inflammation.

## Methods

The protocol for this trial and supporting CONSORT checklist are available as supporting information; see Checklist S1 and Protocol S1.

### Study design

This was a randomized cross-over trial evaluating the acute effects of hypobaric hypoxia at moderate altitude on measures of vascular function, metabolism, sleep, and psychomotor performance in healthy men. The trial was conducted between June and October 2010 and comprised a baseline study in Zurich and an altitude sojourn of 4 days in Davos (Switzerland). Assessments were performed at 3 elevations: 1) Zurich, 490 m, barometric pressure (PB) 958.59 hPa; 2) Davos Wolfgang, 1630 m, PB 839.93 hPa) Davos Jakobshorn, 2590 m (PB 749.27 hPa). Assessments of vascular function and metabolism were performed at 490 m as baseline and daily during two following days at 2590 m. The trials' outcomes on sleep, vigilance and psychomotor performance will be reported elsewhere. The trial was performed and analysed according to CONSORT guidelines (www.consort-statement.org).

### Participants and ethical approval

Subjects were eligible if they were healthy males, between 18 and 70 years old, and living below 800m. Subjects with a body mass index (BMI) <18 or >30 kg/m^2^, any medical condition requiring treatment, regular use of medications, alcohol, nicotine or drugs, a history of sleep disorders or previous altitude related illness at altitude <3000 m were excluded.

The study was approved by the local Zurich ethics committee (KEK ZH 2010–0054/1) and registered (NCT01130948). Written informed consent was obtained from all participants.

The experiments were conducted according to the standards of the Declaration of Helsinki.

### Interventions

**Figure 1:**
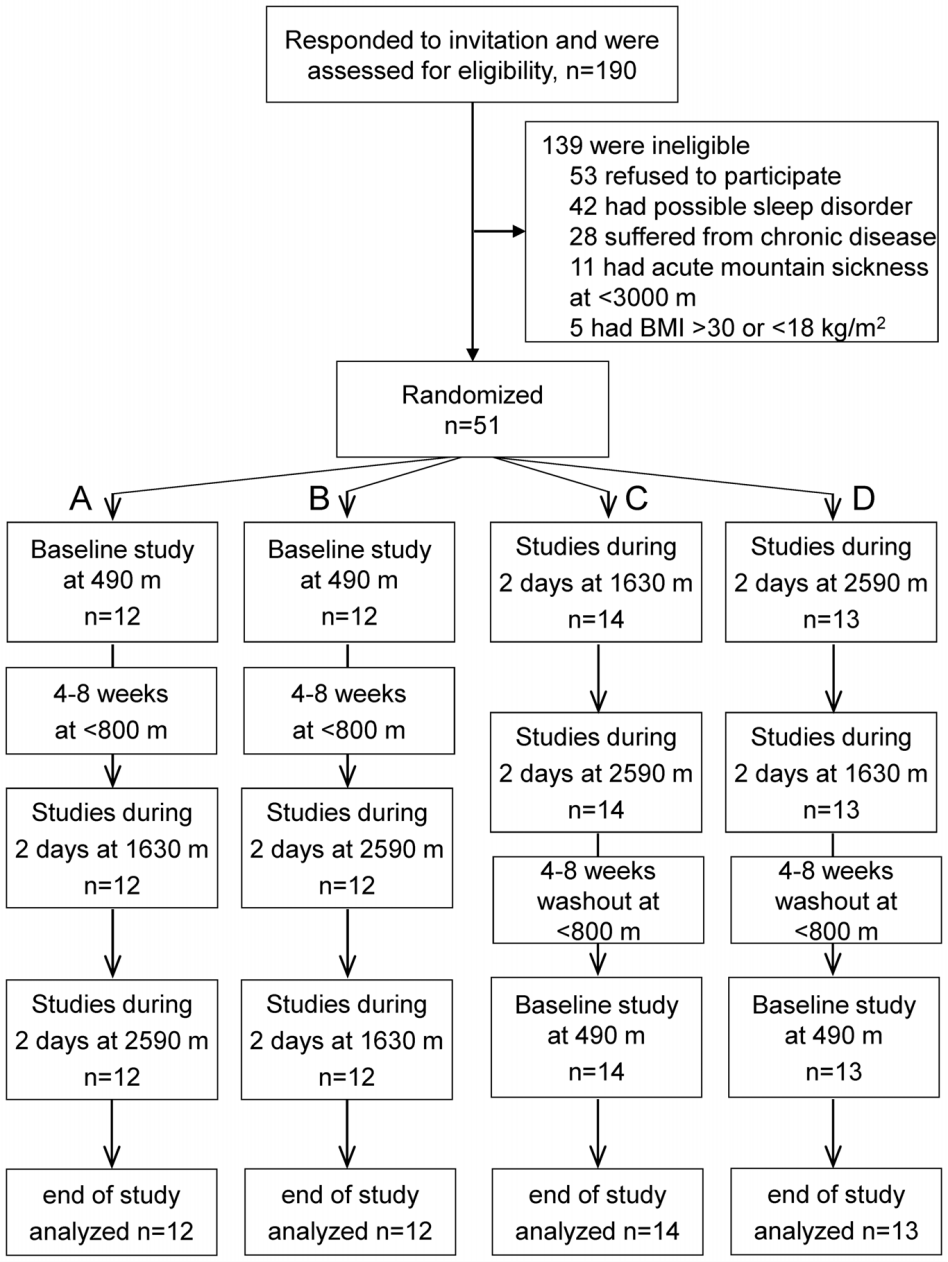
Study flow.

Participants were randomized to 4 groups with permuted sequences of altitude exposure according to a balanced block design: group A) 490 m/1630 m/1630 m/2590 m/2590 m, B) 490 m/2590 m/2590 m/1630 m/1630 m, C) 1630 m/1630 m/2590 m/2590 m/490 m, D) 2590 m/2590 m/1630 m/1630 m/490 m (figure 1). The participants travelled by train from Zurich to Davos and in 30 minutes by cable car from Davos train station (1630 m) to Davos Jakobshorn (2590 m).

### Assessments

All examinations on cardiovascular and metabolic outcomes reported in this manuscript have only been collected at 490 m and 2590 m. On study days all participants were asked to abstain from sporting activities and received standard meals with an energy content of 600–800 kcal consisting of approximately 50% carbohydrates, 30% protein and 20% fat three times daily (at all altitudes). All daytime examinations were performed after waking in the morning after an overnight fast. All clinical measurements were performed by trained physicians. Laboratory and statistical analyses were performed by personnel blinded to randomisation, the altitude and clinical data.

#### Blood pressure, heart rate and digital volume pulse analysis

Blood pressure and heart rate were measured in triplicate in the morning with a standard digital automatic monitor (M6, Omron Healthcare Company, Kyoto, Japan) in supine position after a period of rest of 5 minutes. The average of the three blood pressure measurements was used for further analysis.

Arterial stiffness was estimated by digital volume pulse (DVP) analysis as previously described and validated (Pulse Trace® PCA 2, CareFusion, Rolle, Switzerland) [10]. The device collects 6 representative pulse waveforms with a pulse interval close to the average pulse interval. From the averaged pulse waveforms the arterial stiffness index (SI) is derived, which is defined as height divided by the time from the systolic to diastolic inflection point. Two measurements were performed and the average of the two measurements was used for final analysis.

#### Blood markers of systemic inflammation, insulin resistance and lipid profiles

Blood was drawn in the morning between 6–7 am and blood samples were stored at −80°C for later analysis. Measurements of highly-sensitive C-reactive protein (hsCRP), IL-6, IL-10 and tumor necrosis factor (TNF)-α, lipid profiles and insulin resistance by homeostatic model assessment (HOMA) were performed from plasma samples as previously described [11], [12].

The Dade Behring BN method (particle-enhanced immunonephelometry, measuring range 0.18–1150 mg/L) was used to measure hsCRP as previously described and validated [13].

IL-6, IL-10 and TNF-α were measured by ELISA with commercially available kits (BMS213HS and BMS223HS, Bender MedSystems GmbH, Vienna, Austria). The lower limit of detection for IL-6, IL-10 and TNF-α were 0.03 pg/ml, 0.05 pg/ml and 0.13 pg/ml respectively. The intra- and interassay coefficients of variation were 4.9 % and 6.0 %, respectively for IL-6 and 6.8 % and 7.5 % for IL-10, and 8.5 % and 9.8 % for TNF-α. All blood markers of systemic inflammation were measured in duplicate and in the same batch.

Gradient gel electrophoresis was performed at 10–14°C in 2–16% polyacrylamide gradient gels. Gels were subjected to electrophoresis 24 h at 125 V in tris borate puffer (ph 8.3) as previously described [14], [15]. Gels were fixed and stained for lipids in a solution containing oil red O in 60% ethanol at 55°C. Gels were placed on light source and photographed using a Luminescent Image Analyzer (LAS-3000, Fujifilm). Migration distance for each absorbance peak was determined and the molecular diameter corresponding to each peak was calculated from a calibration curve generated from the migration distance of size standards with known diameter including carboxylated latex beads (Duke Scientific, Palo Alto, CA, USA), thyroglobulin, and apoferritin (HMW Std., Pharmacia, Piscataway NJ, USA) having molecular diameters of 38, 17, and 12.2 nm, respectively, and lipoprotein calibrators of previously determined particle size. The coefficient of variation between measurements was 1.3%. Low density lipoprotein subfraction distribution (LDL-I, -IIA, -IIB, -IIIA, -IIIB, -IVA, and IVB) is expressed as percentage of total LDL [15].

### Outcomes and sample size

Primary outcomes were blood pressure and arterial stiffness index. Additional outcomes were heart rate, blood markers of systemic inflammation, insulin resistance and lipid profiles. To detect clinically relevant differences in mean blood pressure of 5.0 mmHg (SD 6.0) and in stiffness index of 1.0 m/s (SD 1.2) with a two-sided significance level of 0.05 and a power of 80%, a sample size of 46 subjects was necessary [10], [16], [17].

### Data analysis

All values are presented as mean (SD) or median (quartiles) as appropriate. Statistical analyses were performed with Statistica V6.0 (StatSoft, Tulsa, Oklahoma, USA).

Changes in the measured variables from Zurich (490 m) to Davos Jakobshorn (2590 m) were assessed by ANOVA for repeated measurements with Fisher post hoc analysis or non- parametric Friedman ANOVA and Wilcoxon post hoc analysis as appropriate. Statistical significance was assumed at a probability of p<0.05 applying a Bonferroni correction.

## Results

### Subjects

51 male subjects were eligible for entry, were randomised and completed the study protocol (figure 1). The mean age of the study population was 26.9 (9.3) years and the mean body mass index was 23.1 (2.5) kg/m^2^.

### Effects of moderate altitude on oxygen saturation and vascular function

As expected oxygen saturation was significantly lower on both days at 2590 m (table 1) and blood pressure and heart rate were significantly higher compared to 490 m (table 1). Arterial stiffness as assessed by DVP analysis did not significantly change upon ascent to moderate altitude (table 1).

**Table 1:** Effects of moderate altitude on oxygen saturation and vascular function.

Variable	490 m n = 51	2590 m (day1) p vs 490m* n = 51		2590 m (day2) p vs 490 m* n = 51	
Oxygen saturation (%)	96.0 (1.0)	91.0 (2.0)	<0.001	92.0 (2.0)	<0.001
Systolic BP (mmHg)	119.5 (8.6)	123.5 (7.6)	<0.001	125.0 (6.8)	<0.001
Diastolic BP (mmHg)	70.8 (5.1)	74.6 (5.7)	<0.001	75.9 (5.5)	<0.001
Mean BP (mmHg)	87.5 (5.5)	90.9 (5.7)	<0.001	92.3 (6.0)	<0.001
Heart rate (bpm)	56.7 (8.8)	60.1 (10.4)	0.004	60.1 (9.5)	<0.001
Stiffness Index (m/s)	6.2 (1.4)	6.3 (1.3)	NA	6.1 (1.0)	NA

Values are means (SD). BP = blood pressure. * P-values refer to post-hoc comparisons of variables with P ANOVA <0.05. NA: P ANOVA >0.05.

### Effects of moderate altitude on lipid profiles

**Figure 2:**
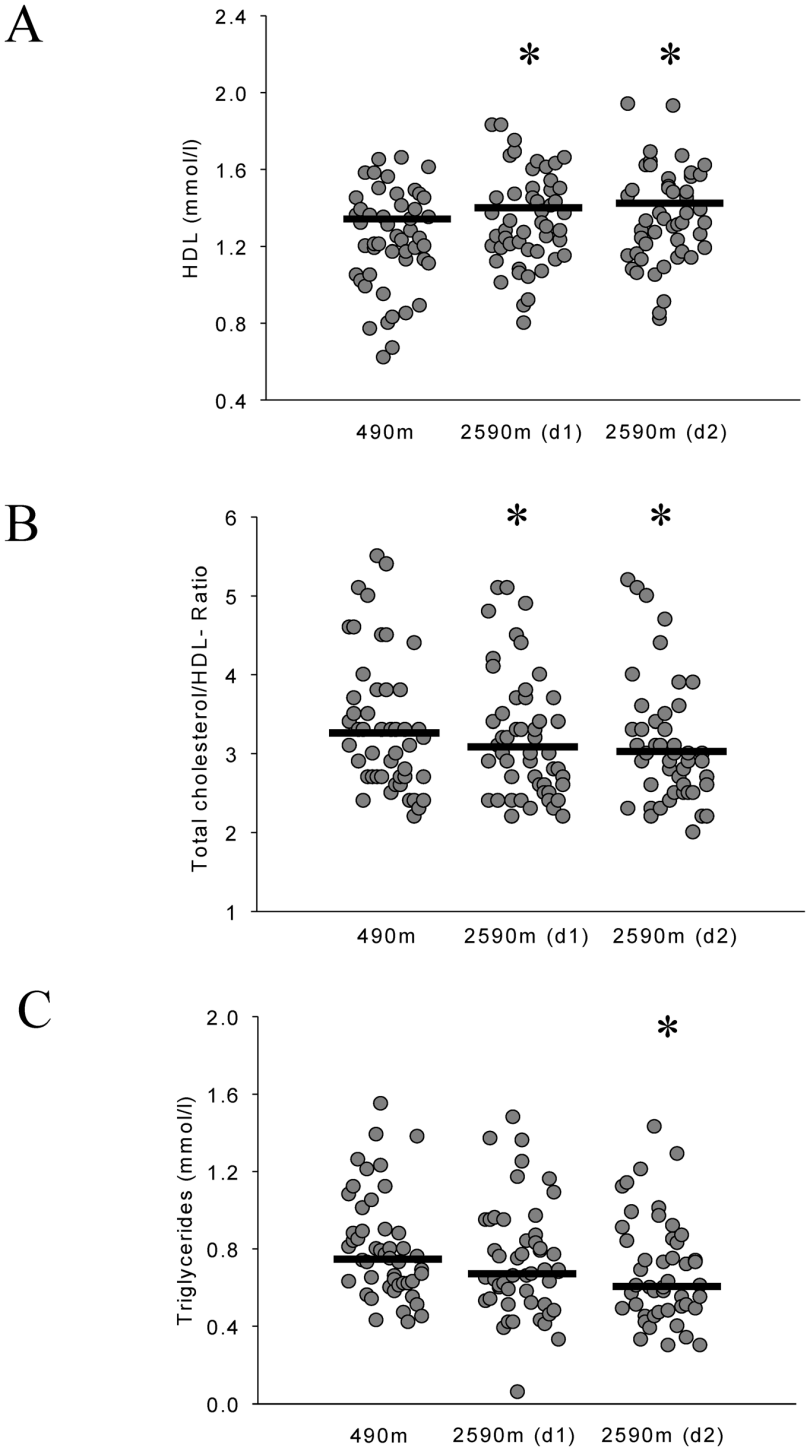
Effects of moderate altitude on lipids. Black lines represent medians. Panel A shows individual high density lipoprotein (HDL) blood levels in Zurich (490 m) and Davos Jakobshorn (2590 m). HDL was higher on both days (d1, d2) at 2590 m compared to 490 m. *p<0.001 vs 490 m. Panel B shows individual total cholesterol/high density lipoprotein (HDL) ratios in Zurich (490 m) and Davos Jakobshorn (2590 m). Total cholesterol/HDL ratio was lower on both days (d1, d2) at 2590 m compared to 490 m. *p<0.05 vs 490 m. Panel C shows individual triglyceride blood levels in Zurich (490 m) and Davos Jakobshorn (2590 m). Triglycerides were lower on day 2 at 2590 m compared to 490 m. *p<0.05 vs 490 m.

**Figure 3:**
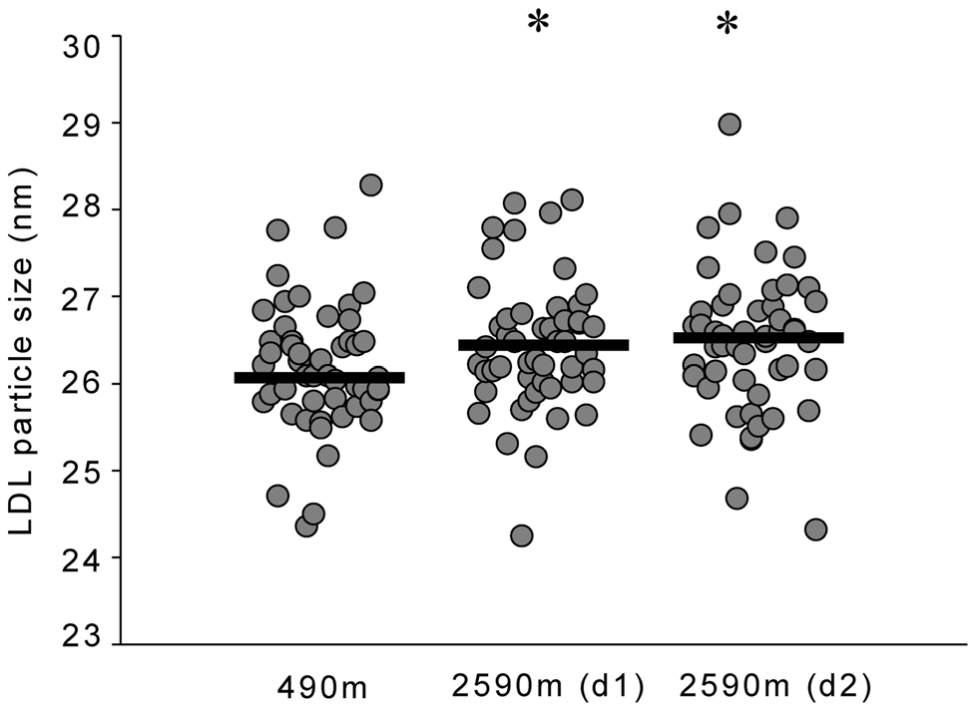
Effects of moderate altitude on LDL diameter. Scatterplot showing individual median low density lipoprotein (LDL) diameter in Zurich (490 m) and Davos Jakobshorn (2590 m) during day 1 and day 2. Black lines represent medians. LDL diameter was larger on both days at 2590 m compared to 490 m. *p<0.05 vs 490 m.

There was no significant change in total and LDL cholesterol upon ascent to 2590 m (table 2). However, HDL cholesterol was significantly higher and total cholesterol/HDL ratio as well as triglyceride levels were significantly lower at 2590 m compared to 490 m (table 2, figure 2). LDL cholesterol particle diameter was significantly greater on both days at 2590 m compared to 490 m (table 2, figure 3). There was an increase of LDL subfraction I upon ascent to 2590 m (table 2).

**Table 2:** Effects of moderate altitude on metabolism and inflammation.

Variable	490 mn = 51	2590 m (day 1) n = 51	P vs 490m*	2590 m (day 2) n = 51	P vs 490m*
Triglycerides (mmol/l)	0.75 (0.62, 0.9)	0.67 (0.52, 0.9)	0.094	0.61 (0.49, 0.9)	0.012
Total Chol (mmol/l)	3.70 (3.6, 4.4)	4.0 (3.70, 4.5)	NA	4.0 (3.5, 4.5)	NA
HDL Chol (mmol/l)	1.25 (1.1, 1.41)	1.3 (1.18, 1.5)	<0.001	1.33 (1.16, 1.53)	<0.001
Total Chol/HDL-Ratio	3.2 (2.7, 3.8)	3.0 (2.6, 3.7)	0.015	2.95 (2.5, 3.4)	0.001
LDL Chol (mmol/l)	2.2 (2.0, 2.8)	2.4 (2.0, 2.8)	NA	2.2 (1.9, 2.6)	NA
LDL particle size (nm)	26.08 (25.8, 26.5)	26.37 (26.01, 26.71)	0.008	26.53 (26.03, 26.9)	0.048
LDL I (%)	18.8 (16.3, 23.3)	22.2 (18.1, 26.3)	0.009	21.7 (19.0, 26.1)	0.022
LDL IIA (%)	14.8 (12.7, 16.3)	15.1 (13.0, 16.4)	NA	15.3 (13.7, 17.1)	NA
LDL IIB (%)	20.0 (18.0, 24.1)	18.9 (16.3, 22.7)	NA	19.4 (16.1, 21.5)	NA
LDL IIIA (%)	16.5 (14.7, 19.3)	16.2 (13.6, 17.8)	NA	15.4 (13.3, 18.0)	NA
LDL IIIB (%)	6.4 (5.6, 7.6)	6.0 (5.5, 7.3)	NA	5.9 (5.1, 7.1)	NA
LDL IVA (%)	9.5 (8.4, 10.2)	8.9 (9.8, 7.8)	NA	9.0 (8.0, 10.3)	NA
LDL IVB (%)	10.6 (9.3, 12.3)	10.1 (12.1, 9.1)	NA	11.1 (9.4, 12.6)	NA
hsCRP (mg/l)	0.44 (0.18, 0.91)	0.26 (0.17, 0.53)	0.024	0.33 (0.17, 0.5)	0.054
Interleukin-6 (pg/ml)	0.54 (0.28, 0.94)	0.40 (0.26, 0.62)	NA	0.43 (0.28, 0.63)	NA
Interleukin-10 (pg/ml)	0.51 (0.36,1.2)	0.56 (0.31, 1.1)	NA	0.64 (0.34, 0.92)	NA
TNF-α (pg/ml)	0.77(0.67, 1.0)	0.83 (0.62, 1.0)	NA	0.77 (0.63, 1.04)	NA
Glucose (mmol/l)	5.2 (5.0, 5.4)	5.1 (5.0, 5.4)	NA	5.1 (4.9, 5.4)	NA
Insulin (μU/ml)	2.0 (2.0, 2.8)	2.0 (2.0, 3.1)	NA	2.0 (2.0, 2.2)	NA
HOMA-Index	0.47 (0.44, 0.7)	0.46 (0.44, 0.71)	NA	0.47 (0.44, 0.7)	NA

Values are medians (quartiles). Chol = cholesterol; HDL =  high density lipoprotein; LDL =  low density lipoprotein, LDL I, IIA, IIB, IIIA, IIIB, IVA, IVB = LDL subfractions. HsCRP =  highly sensitive C-reactive protein, TNF-α =  Tumor necrosis factor α. HOMA-index =  Homeostatic model assessment-index = fasting insulin (μU/ml) x fasting glucose (mmol/l)/22.5. *P-values refer to post-hoc comparisons of variables with P ANOVA <0.05. NA: P ANOVA > 0.05.

### Effects of moderate altitude on systemic inflammation

**Figure 4:**
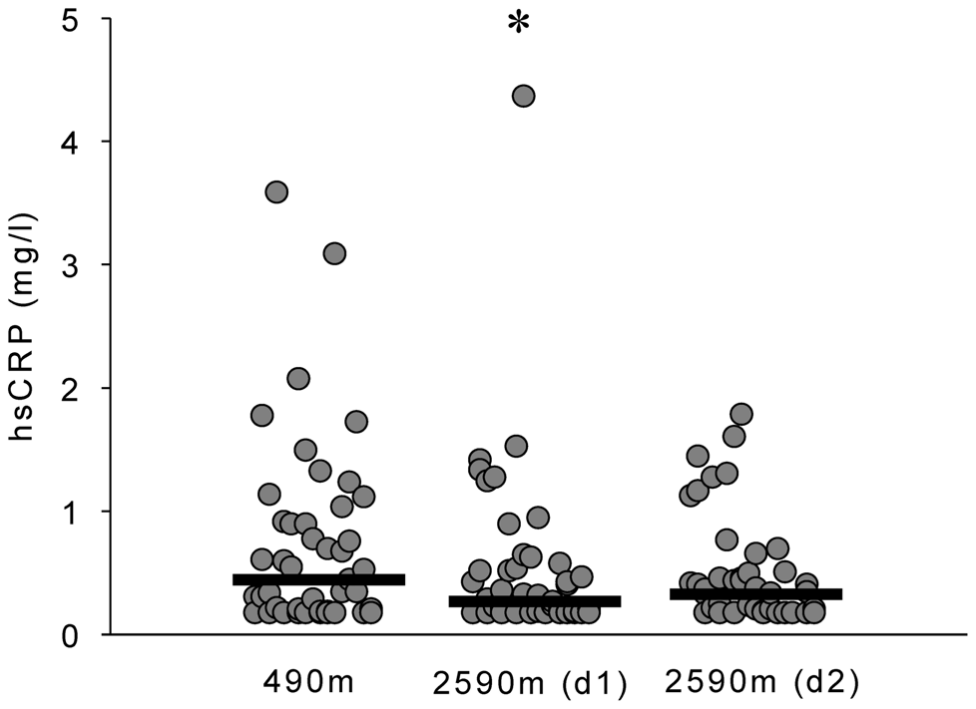
Effects of moderate altitude on C-reactive protein. Scatterplot showing individual highly sensitive C-reactive protein (hsCRP) blood levels in Zurich (490 m) and Davos Jakobshorn (2590 m). Black lines represent medians. HsCRP levels were lower on day 1 at 2590 m compared to 490 m. *p<0.05 vs 490 m.

HsCRP decreased significantly upon ascent to 2590 m (table 2, figure 4), however, no statistically significant changes in IL-6, IL-10 and TNF-α were observed (table 2).

### Effects of moderate altitude on glucose metabolism

There was no significant change in blood glucose, insulin and insulin resistance as assessed by HOMA upon ascent to 2590 m (table 2).

## Discussion

This is the first randomised controlled study to investigate the effects of acute exposure to moderate altitude on vascular function, glucose and lipid metabolism as well as on systemic inflammation in healthy males. We found that exposure to moderate altitude was associated with significant increases in blood pressure and heart rate, possibly due to augmented sympathetic activity. Short-term stay at moderate altitude was associated with improvements in lipid profiles and a decrease in systemic inflammation. However, moderate altitude did not have a significant effect on glucose metabolism.

In the current study ascent to 2590 m, an altitude commonly visited by families during skiing and hiking vacations, resulted in hypoxemia in all subjects (mean SpO_2_ 91%). Exposure to mild hypobaric hypoxia was associated with considerable increases in both blood pressure and heart rate (table 1). This is in contrast to a small study including 10 normotensive subjects in which no effect of acute exposure to moderate altitude (2572 m) on blood pressure was found [1]. A possible explanation for this discrepancy is the small sample size of the study by D'Este and colleagues [1] (10 normotensive and 13 hypertensive subjects) as well as differences regarding the population studied. However, the findings of studies investigating the effects of high and very high altitude in healthy subjects suggest that acute exposure to more severe hypobaric hypoxia is associated with increased sympathetic activity resulting in elevated heart rate and blood pressure [18]–[21]. The observed acute effects of moderate altitude on blood pressure and heart rate may be even more pronounced in patients with arterial hypertension [19], and may possibly add to the peak of risk for myocardial infarction observed within the first two days of vacation in patients with coronary artery disease [22], [23].

Despite the observed increase in blood pressure and heart rate, arterial stiffness as estimated by digital volume pulse analysis derived stiffness index (SI) did not change significantly at moderate altitude in the current study. This is concordant with the report of Rhodes et al. [24] who found an unchanged SI in 17 subjects after ascent to 3450 m. Thus it seems that acute exposure to moderate and high altitude has no immediate effect on arterial stiffness.

Upon ascent to moderate altitude lipid profiles improved significantly in our study, i.e. a decrease in triglycerides and total cholesterol/HDL ratio as well as an increase in HDL was observed (table 2). Moreover, at altitude the average LDL particle size increased, which was related to a significant increase in LDL subfraction I particles (figure 3). These changes are consistent with a reduction of atherogenicity and are thus thought to reduce cardiovascular risk [25], [26]. Low-density lipoprotein (LDL) size is an important predictor of cardiovascular events and progression of coronary artery disease, and the predominance of small, dense LDL has been accepted as an emerging cardiovascular risk factor in various populations. Since the therapeutic modulation of distinct LDL subspecies, as seen in our trial, seems to result in reduced cardiovascular events [8], [26], [27], our findings suggest that mild hypobaric hypoxia may be beneficial to modulate atherogenicity. To our knowledge there is no other data on the acute effects of moderate altitude on lipid profiles and LDL particle size in healthy humans. The results from studies investigating the effects of high and very high altitude on blood lipids are contradictory [28]–[30], possibly due to small sample sizes, heterogeneity in the study populations and differences related to the level of altitude. However, the findings of a study investigating the effects of a hiking vacation at moderate altitude (1700 m) in patients with the metabolic syndrome suggest that activity at moderate altitude is associated with improvement of lipid profiles after 3 weeks [2], [4].

These effects may be sustained, as it has been found in epidemiological studies that HDL levels increase when living at higher altitude [31].

We observed no significant effect of acute exposure to moderate altitude on various measures of glucose metabolism in healthy humans. This is in contrast to a Chinese study which reported improvement of glucose tolerance as assessed by oral glucose tolerance testing (OGTT) after 3 days at 2400 m in 9 untrained subjects [3]. This discrepancy may be explained by the small sample size, the different method used to assess glucose metabolism, the different ethnicity and other differences regarding the participant characteristics in the study by Lee et al [3]. However, the lack of an effect on HOMA in our study suggests that there is no clinically important effect of acute exposure to moderate altitude on insulin resistance in healthy Caucasians.

There is some evidence that severe hypobaric, hypoxic conditions at high altitude stimulate the expression of pro-inflammatory markers such as hsCRP and IL-6 [32]–[34]. In the current study, acute exposure to moderate altitude was associated with a significant decrease in systemic inflammation as assessed by hsCRP, but no change in IL-6, IL-10 or TNF-α was observed. To our knowledge there are no other data published on the effect of moderate altitude on systemic inflammation. Our findings suggest that acute, mild hypobaric-hypoxia has an anti-inflammatory effect. In contrast severe hypobaric hypoxia experienced at higher altitudes seems to have a pro-inflammatory effect. The divergent findings of previous studies regarding the effect of altitude on hsCRP can possibly be explained by different altitude levels (moderate vs high altitude), differences of the studied populations (healthy vs diseased participants), the non-randomised design and small sample size of previous studies, and differences of physical activity levels during the study period.

Our study has some limitations; as it was designed to assess the short-term effects of moderate altitude exposure, we do not know whether the observed effects on the cardiovascular system, lipid metabolism and systemic inflammation are sustained during a longer stay at moderate altitude; a question that needs to be addressed in future studies. As we selectively included healthy male subjects to study the effects of moderate altitude in a large homogeneous population, the findings of the current study should be extrapolated with caution to females and patients with cardiovascular or metabolic disease. Although it is conceivable that similar effects can be expected in females and such patients, this will need to be proven in further studies.

In conclusion, the current study has shown that a short-term stay at moderate altitude is associated with mild hypoxemia leading to increased blood pressure and heart rate, likely due to augmented sympathetic activity. In contrast, exposure to moderate altitude seems to improve the lipid profile and systemic inflammation, however, it seems to have no relevant effect on glucose metabolism.

## Supporting Information

Checklist S1 **CONSORT Checklist.**
(DOC)

Protocol S1 **Trial Protocol.**
(PDF)
