# Supplementary material for: Effects of Acute Exposure to Moderate Altitude on Vascular Function, Metabolism and Systemic Inflammation
Source: PLoS One. 2013 Aug 1;8(8):e70081. doi: 10.1371/journal.pone.0070081 (PMC3731354; doi:10.1371/journal.pone.0070081)
Supplement: Protocol S1 — Trial Protocol. (PDF) [file pone.0070081.s002.pdf]

UniversitätsSpital Zürich

Universität Zürich

Sleep, breathing and psychomotor performance at altitude: A physiologic study in healthy subjects

Protocol ID: 2010-0054

Version 2.0

04.05.2010

Amendments from 04.05.2010 are marked with a diagonal pattern box

Correspondence: Prof. Dr. med. Konrad E. Bloch
Klinik für Pneumologie
Universitätsspital Zürich
RAE-C28
Rämistrasse 100
CH-8091 Zürich
konrad.bloch@usz.ch
Tel: 044 255 3828
Fax: 044 255 4451

Table of Contents

1 GENERAL INFORMATION 4

2 STUDY SYNOPSIS 6

3 STUDY FLOW CHART 9

3.1 TIME TABLE 9
3.2 STUDY FLOW CHART 9

4 LIST OF ABBREVIATIONS 10

5 INTRODUCTION 11

5.1 BACKGROUND 11
5.2 SIGNIFICANCE OF THE PROJECT 12
5.3 RATIONALE FOR CURRENT STUDY 12
5.3.1 Hypotheses 12

6 STUDY OBJECTIVES 13

6.1 PRIMARY OBJECTIVES 13
6.2 ADDITIONAL OBJECTIVES 13

7 STUDY DESIGN 13

7.1 DETAILED STUDY DESIGN FOR THE STUDY PHASE 1 EVALUATING THE EFFECT OF ALTITUDE 13
7.1.1 Schedule of daily assessments study phase 1 15
7.2 DETAILED STUDY DESIGN FOR THE STUDY PHASE 2 EVALUATING THE COMBINED EFFECTS OF SLEEP RESTRICTION AND ALTITUDE 16
7.2.1 Schedule of daily assessments study phase 2, normal sleep 18
7.2.2 Schedule of daily assessments study phase 2, restricted sleep 19
7.3 STUDY OUTCOME MEASURES 19
7.3.1 Primary outcomes 19
7.3.2 Additional outcomes 19

8 PARTICIPANT ENTRY 21

8.1 PRE-REGISTRATION EVALUATIONS 21
8.2 INCLUSION CRITERIA 21
8.3 EXCLUSION CRITERIA 21
8.4 WITHDRAWAL CRITERIA 21

9 EFFICACY AND SAFETY VARIABLES 22

9.1 EFFICACY VARIABLES 22
9.1.1 Symptom evaluation 22
9.1.2 Sleep and breathing 22
9.1.3 Vigilance and psychomotor performance 22
9.1.4 Cardiovascular function 23

9.2 SAFETY VARIABLES 24

10 ADVERSE EVENTS 25

10.1 DEFINITIONS 25
10.1.1 Adverse Event (AE): 25
10.1.2 Serious Adverse Event (SAE): 25
10.2 REPORTING PROCEDURES 26
10.2.1 Non serious AEs 26
10.2.2 Serious AEs 26

11 ASSESSMENT AND FOLLOW-UP 27

11.1 RECRUITMENT 27
11.2 DAY 1-5 (PHASE 1) AND DAY 1-8 (PHASE 2) 27
11.3 FOLLOW-UP 27

12 STATISTICS AND DATA ANALYSIS 27

13 REGULATORY ISSUES 28

13.1 ETHICS APPROVAL 28
13.2 CONSENT 28
13.3 CONFIDENTIALITY 28
13.4 INSURANCE 28
13.5 FUNDING 28
13.6 AUDITS 28

14 STUDY MANAGEMENT 29

14.1 DATA MANAGEMENT 29

15 PUBLICATION POLICY 29

16 REFERENCE LIST 30

17 APPENDICES 33

18 DECLARATIONS OF SPONSOR-INVESTIGATOR AND INVESTIGATOR 34

18.1 DECLARATION OF SPONSOR-INVESTIGATOR 34
18.2 DECLARATION OF INVESTIGATOR 34

1 GENERAL INFORMATION

**Sponsor-Investigator and Principal Investigator:**	Prof. Dr. med. Konrad E. Bloch<br>Klinik für Pneumologie,<br>Universitätsspital Zürich<br>Rämistr. 100, 8091 Zürich<br>Tel.: 044 255 38 28<br>Fax.: 044 255 44 51<br>Email: konrad.bloch@usz.ch
**Investigator(s):**	Dr. med. Tsogyal Latshang,<br>Klinik für Pneumologie,<br>Universitätsspital Zürich<br>Rämistr. 100, 8091 Zürich<br>Tel.: 044 255 38 28<br>Fax.: 044 255 44 51<br>Email: tsogyal.latshang@usz.ch<br><br>Dr. med. Christian Lo Cascio<br>Klinik für Pneumologie,<br>Universitätsspital Zürich<br>Rämistr. 100, 8091 Zürich<br>Tel.: 044 255 38 28<br>Fax.: 044 255 44 51<br>Email: christian.locascio@usz.ch<br><br>Katrin Stadelmann, Msc<br>Institut für Pharmakologie u. Toxikologie,<br>Winterthurerstrasse 190<br>CH-8057 Zürich<br>Tel. +044 635 59 63<br>Fax.: 044 635 57 07<br>Email: k.stadelman@pharma.uzh.ch<br><br>PD Dr. med. Malcolm Kohler,<br>Klinik für Pneumologie,<br>Universitätsspital Zürich<br>Rämistr. 100, 8091 Zürich<br>Tel.: 044 255 38 28<br>Fax.: 044 255 44 51<br>Email: malcolm.kohler@usz.ch<br><br>Prof. Dr. sc. nat. Peter Achermann<br>Institut für Pharmakologie u. Toxikologie,<br>Winterthurerstrasse 190

	CH-8057 Zürich<br>Tel. +044 635 59 54<br>Fax. +044 635 57 07<br>Email: acherman@pharma.uzh.ch<br><br>Prof. Dr. sc. nat. Reto Huber,<br>Forschungsleiter,<br>Interdisziplinäres Zentrum für Schlafmedizin<br>Kinderspital Zürich,<br>Steinwiesstrasse 75,<br>8032 Zürich<br>Tel.: 044 266 81 60,<br>Fax.: 044 255 71 63<br>Email: reto.huber@kispi.uzh.ch
**Study monitor:**	Clinical Trial Centre<br>UniversitätsSpital Zürich<br>Rämistr. 100, 8091 Zürich<br>Tel: +41 (0)44 634 55 06<br>Fax: +41 (0)44 634 55 03
**Study site:**	Klinik für Pneumologie,<br>Universitätsspital Zürich<br>Rämistr. 100, 8091 Zürich
**Laboratory and other institutions involved in the trial :**	Hochgebirgsklinik Davos<br>Hermann Burchard Strasse1<br>CH-7265 Davos-Wolfgang<br>Tel. 081-417 44 44<br>Fax 081-417 30 30<br><br>Hotel Jakobshorn<br>CH-7265 Davos<br>Tel: 081 413 70 04<br>Fax: 081 413 19 64

2 STUDY SYNOPSIS

**Study Title:**	Sleep, breathing and psychomotor performance at altitude: A physiologic study in healthy subjects
**Protocol Version and Date:**	Protocol ID: 2010-0054<br>Version: 2.0<br>Amendment # 1<br>Date 04.05.2010
**Methodology:**	Physiologic study evaluating effects of short-term exposure to moderate altitude without and with sleep restriction on sleep, breathing, pulmonary function and psychomotor performance in healthy subjects. A randomized cross-over design is used. Outcomes are assessed by noninvasive techniques. Study locations include the University Hospital of Zurich (490m) and 2 moderate altitudes locations representative for many alpine resorts, i.e., Davos Wolfgang (Höhenklinik at 1631m) and Jakobshorn (Hotel at 2590m). The study is performed in two phases. In phase 1, the effect of altitude exposure is investigated; in phase 2, combined effects of altitude and sleep restriction are investigated.
**Study Duration:**	Study phase 1 is performed in summer 2010 (June-October). Total study duration for each participant is 6 days (2 days in Zurich, 4 days at altitude).<br>Study phase 2 is performed in summer 2011 (June-October). Total study duration for each participant is 9 days (5 days in Zurich, 4 days at altitude).<br>If study objectives are not achieved during summer 2010/11, complementary measurements will be performed in 2012.
**Study Center:**	Klinik für Pneumologie,<br>Universitätsspital Zürich<br>Rämistr. 100, 8091 Zürich

**Investigator(s):**	Prof. Dr. med. Konrad E. Bloch<br>Klinik für Pneumologie, Universitätsspital Zürich<br>Rämistr. 100, 8091 Zürich<br>Tel.: 044 255 38 28, Fax.: 044 255 44 51<br>Email: konrad.bloch@usz.ch<br><br>Dr. med. Tsogyal Latshang<br>Klinik für Pneumologie, Universitätsspital Zürich<br>Rämistr. 100, 8091 Zürich<br>Tel.: 044 255 38 28, Fax.: 044 255 44 51<br>Email: tsogyal.latshang@usz.ch<br><br>Dr. med. Christian Lo Cascio<br>Klinik für Pneumologie, Universitätsspital Zürich<br>Rämistr. 100, 8091 Zürich<br>Tel.: 044 255 38 28, Fax.: 044 255 44 51<br>Email: Christian.locascio@usz.ch<br><br>PD Dr. med. Malcolm Kohler<br>Klinik für Pneumologie, Universitätsspital Zürich<br>Rämistr. 100, 8091 Zürich<br>Tel.: 044 255 38 28, Fax.: 044 255 44 51<br>Email: malcolm.kohler@usz.ch<br><br>Prof. Dr. sc. nat. Peter Achermann<br>Institut für Pharmakologie und Toxikologie,<br>Winterthurerstrasse 190, CH-8057 Zürich<br>Tel. +044 635 59 54, Fax.: 044 635 57 07<br>Email: acherman@pharma.uzh.ch<br><br>Prof. Dr. sc. nat. Reto Huber<br>Forschungsleiter, Interdisziplinäres Zentrum für<br>Schlafmedizin, Kinderspital Zürich,<br>Steinwiesstrasse 75, 8032 Zürich<br>Tel.: 044 266 81 60, Fax.: 044 255 71 63<br>Email: reto.huber@kispi.uzh.ch<br><br>Katrin Stadelmann, Msc.<br>Inst. für Pharmakologie und Toxikologie,<br>Winterthurerstrasse 190, CH-8057 Zürich<br>Tel. 044 635 59 63, Fax.: 044 635 57 07<br>Email: k.stadelman@pharma.uzh.ch

**Objective(s):**	Primary study objectives:<br>Phase 1<br>To evaluate the effect of short-term exposure to moderate altitude on sleep related breathing disturbances, sleep quality, psychomotor performance, pulmonary function and cardiovascular function.<br>Phase 2<br>To evaluate the combined effect of short-term exposure to moderate altitude and sleep restriction on sleep related breathing disturbances, sleep quality, psychomotor performance and cardiovascular function.
**Number of Subjects:**	Based on the sample size estimation 52 healthy subjects will be included in each study phase, which means 52 in 2010 and 52 in 2011.
**Diagnosis and Main Inclusion Criteria:**	- Healthy subjects<br>- Age 20-70 y<br>- Male gender (inclusion of females is not feasible because it would require to control for the effects of the menstrual cycle on ventilation and various other physiological processes)<br>- Informed consent.
**Main Exclusion Criteria:**	- Any medical condition requiring treatment<br>- Regular use of medications, nicotine or drugs<br>- History of sleep apnea, sleep disturbances or previous high altitude related illness at <3000m (acute mountain sickness, high altitude cerebral or pulmonary edema requiring unplanned descent, medical treatment or evacuation).<br>- salbutamol-/nitroglycerin-intolerance<br>- BMI <18 or >30 kg/m^2
**Study Schedule:**	01/2010 through 12/2012
**Statistical Methodology:**	-Graphical display of raw data, plausibility checks, evaluation of data distribution<br>-Descriptive statistics<br>-Evaluation of the effects of altitude and sleep restriction by ANOVA and by multiple regression analysis
**Statement:**	This study will be conducted in compliance with the protocol, the current version of the Declaration of Helsinki, and ICH-GCP as well as all national legal and regulatory requirements. Collection of human biological material will also follow the SAMW Guidelines.

3 Study Flow Chart

3.1 TIME TABLE

Table 1. Time table of study

Year	Activity
2010	-months 1-6: preparation of measurement techniques, pilot studies, preparation of field studies
	-months 4-10: subject recruitment and field studies phase 1
	-months 10-12: data analysis
2011	-months 1-6: data analysis, preparation of field studies
	-months 4-10: subject recruitment and field studies phase 2
	-months 10-12: data analysis, publication
2012	-months 1-3: data analysis
	-months 4-10: complementary measurements if required
	-months 6-12: study completion, data analysis, publication

3.2 STUDY FLOW CHART

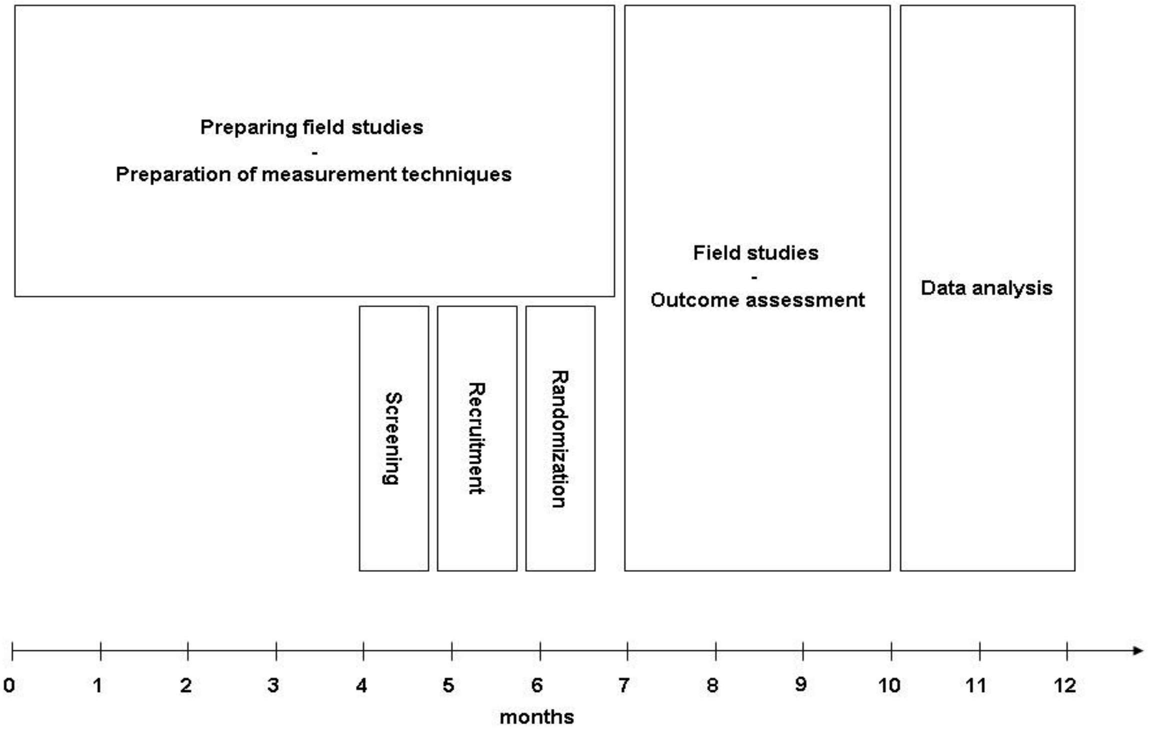

Figure 1: Chart of activities during the 12 months in each year

4 List of Abbreviations

AE	Adverse Event
AIx	Augmentation-Index
AMS	Acute Mountain Sickness
AMS–C	Cerebral symptoms of AMS
BMI	Body Mass Index
CPAP	Constant Positive Airway Pressure
CRF	Case Report Form
DADS	Divided Attention Steering Simulator
ECG	Electrocardiography
EEG	Electroencephalography
EMG	Electromyelography
EOG	Electrooculography
ESS	Epworth sleepiness scale
ESQ	Enviromental symptoms questionnaire
GCP	Good Clinical Practice
HAPE	High Altitude Pulmonary Edema
HMG	Heilmittelgesetz
ICH	International Conference on Harmonisation
IEC	Independent Ethics Committee
MSLT	Multiple sleep latency test
MTM	Motor Task Manager
OSA	Obstructive sleep apnoea
SDQ	Sleep Disorder Questionnaire
PVT	Psychomotor Vigilance Test
Vklin	Verordnung über klinische Versuche

5 INTRODUCTION

5.1 BACKGROUND

Sleep is essential for well being, mood, vigilance, cognitive performance and learning[1;2] . Sleep influences various physiological processes including functions of the respiratory and cardiovascular systems, the brain and the autonomic nervous system, among others[1]. Healthy subjects may experience sleep disturbances associated with periodic breathing when exposed to hypoxia at altitude[2;3] and there is evidence that both intermittent and sustained hypoxia at altitude[4] induce autonomic cardiovascular dysfunction with increased oscillatory and tonic sympathetic output. Altitude related illness including acute mountain sickness (AMS) and subclinical or overt high altitude pulmonary edema may additionally interfere with sleep, breathing and central and autonomic nervous system function in un-acclimatized visitors to mountain areas [2]. There are concerns that hypoxia not only affects sleep, ventilation and cardiovascular function, but also impairs cognitive and psychomotor performance [5;6]. These adverse effects may be particularly deleterious if combined with sleep restriction which itself causes impaired cognitive performance and emotional stress [7]. This may be relevant in tourists arriving at altitude late in the evening and getting up early the next morning after a short sleep of poor quality engaging in activities that are potentially dangerous for themselves and others such as driving a vehicle[8], skiing or snow boarding. Supporting these concerns, we observed that patients with the obstructive sleep apnea syndrome performed poorly during a temporary stay at Davos (2590m) in simulated driving, revealed high blood pressure and frequent cardiac arrhythmia in comparison to baseline measurements in Zurich (490m)[9].

Although many persons, some even with respiratory or cardiac disease, travel to high altitude for professional and leisure activities the effects of hypoxia on sleep, ventilation, cardiovascular function and daytime performance are still not well understood. From a current systematic literature analysis we were able to draw only limited conclusions on sleep at altitude because of the flawed design of many studies that did not control for several confounding factors and due to insufficient statistical powered [10]. Furthermore, sophisticated and sensitive tools for assessing neurophysiologic and cognitive function were not available in earlier studies. Understanding the interactions among hypoxia, sleep and its restriction, cardio-respiratory and cognitive functions is an important requirement for counseling persons travelling to high altitude and involved in activities that depend on physical and mental fitness. Therefore, we wish to further explore the effects of hypoxia. Therefore we will 1) employ a robust study design, 2) an adequate number of participants and 3) novel sophisticated techniques to detect subtle physiologic changes in response to altitude exposure and sleep restriction.

Thus, in addition to a detailed analysis of cardio-respiratory function we will investigate the EEG characteristics of sleep including slow waves. Steriade[11] and co-workers showed that during slow oscillations membrane potentials of cortical neurons alternate about every second between a depolarized up-state and a hyperpolarized down-state. The alternation of up- and down-states in cortical neurons is thought to be involved in memory consolidation [11-13] synaptic homeostasis[13], and the recuperative function of sleep. Thus, the quantification of slow-waves in the sleep EEG using spectral analysis and their morphological characterization reveals objective insights into the brain state under various conditions including hypoxia and sleep restriction. Sophisticated

daytime evaluation of psychomotor performance will provide an objective assessment of the consequences on daytime function.

In healthy subjects, a high prevalence of subclinical pulmonary edema not associated with major symptoms has been observed after rapid ascent to 4559m at Mt. Rosa[A1, A2]. This was been assessed by specialized pulmonary function tests including measurement of diffusing capacity for carbon monoxide. Furthermore, in patients with the obstructive sleep apnea syndrome we have recently observed similar alterations in pulmonary function tests at Davos Jakobshorn (2590m) [A3]. This raises the question whether HAPE occurs even after a rapid ascent to 2590m at Davos Jakobshorn and whether this may affect their physical performance.
A1) Cremona et al. *Lancet* 359 (9303):303-309, 2002. A2.) O. Senn et al.. *Med Sci.Sports Exerc.* 38 (9):1565-1570, 2006. A3.) Latshang et al. Swiss Med Weekly 2010, abstract.

5.2 SIGNIFICANCE OF THE PROJECT

Knowledge on sleep and cognitive performance at altitude is still scant and largely limited to uncontrolled observations. A large number of people are exposed to altitude and there are concerns about unfavourable effects on their health and performance. Professionals using machinery, car drivers and persons engaged in activities at altitude such as mountaineering, skiing or snow boarding are often sleep deprived. This may additionally impair psychomotor performance exposing them and others to a high risk of accidents. Our project aims at closing the gap in knowledge on interactions among hypoxia at altitude, sleep (including sleep restriction and disturbance), cardio-respiratory functions and psychomotor performance by randomized, controlled studies using powerful techniques. Understanding physiologic mechanisms of altitude on sleep and cognitive performance may help to prevent altitude induced impairments and to improve well-being and cognitive performance. By using innovative, sophisticated techniques, both physiologically and clinically relevant outcomes will be evaluated to investigate mechanisms and potential hazards related to altitude exposure.

5.3 RATIONALE FOR CURRENT STUDY

The purpose of this project is to investigate sleep, breathing, psychomotor and cardiovascular function and their interrelation in healthy volunteers exposed to hypoxia during altitude field studies.

5.3.1 Hypotheses

Phase 1: In *healthy subjects* exposure to hypobaric hypoxia at altitude
1. affects sleep in a dose (altitude) dependent way, partly related to periodic breathing.
2. impairs psychomotor performance in correlation with sleep and breathing disturbances.
3. induces sympathetic activation, endothelial dysfunction and alters arterial stiffness.
4. impairs pulmonary function

Phase 2: In *healthy subjects*, combined exposure to hypobaric hypoxia at altitude and sleep restriction
1. differently affects sleep and breathing compared to each stimulus alone.

2. has an additive effect on sympathetic activation, endothelial function and arterial stiffness.
3. has an additive negative effect on psychomotor performance.
4. impairs pulmonary function

6 STUDY OBJECTIVES

6.1 PRIMARY OBJECTIVES

To evaluate the effects of altitude and to evaluate the combined effects of altitude and sleep restriction on

- Breathing during sleep
- Sleep quality
- Vigilance and psychomotor performance
- Cardiovascular function
- Pulmonary function testing

6.2 ADDITIONAL OBJECTIVES

To perform detailed studies on the physiologic effects of altitude and sleep restriction using novel techniques that simulate real life situation.

7 STUDY DESIGN

The project will comprise 2 physiological studies performed in healthy subjects sequentially over the course of 2-3 years. Studies locations will be at the University Hospital of Zurich and in research facilities temporarily installed in Davos at 2 moderate altitudes (1700m and 2590m) representing the altitude range of Alpine settlements and mountain resorts. A randomized, cross-over design will be used to control for order and other confounding effects.

The study will be performed in compliance with the protocol, GCP and the applicable regulatory requirement (HMG, Vklin).

7.1 DETAILED STUDY DESIGN FOR THE STUDY PHASE 1 EVALUATING THE EFFECT OF ALTITUDE

The purpose is to investigate effects of hypobaric hypoxia at altitude on sleep, breathing, psychomotor performance and cardiovascular function. A randomized, cross-over design will be used. After recruitment, individuals will be randomized to one of 4 different sequences of studies as shown in table 2 and figure 2. Evaluations at all locations will be identical.

Table 2. Sequences of evaluation in study phase 1			
Day -60 to 0: Subject screening, recruitment, randomization			
Sequence A	Sequence B	Sequence C	Sequence D
490 m, day 1	490 m, day 1	1630m, day 1-2	2590 m, day 1-2
1631 m, day 2-3	2590 m, day 2-3	2590 m, day 3-4	1631 m, day 3-4
2590 m, day 4-5	1631 m, day 4-5	490 m, day 5	490 m, day 5

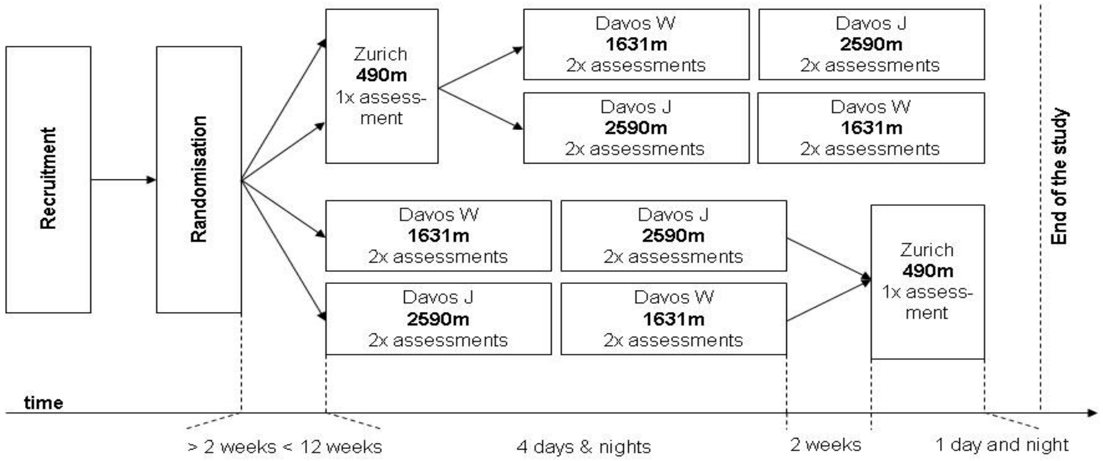

Figure 2: Study phase 1 design representing subject flow and the 4 different sequences of evaluations. Study locations comprise the Clinical Trial Center, University Hospital of Zurich (Zurich, 490m), the Deutsche Hochgebirgs Klinik Davos Wolfgang (Davos W, 1631m) and the Hotel at Jakobshorn, Davos (Davos J, 2590m). At all locations single room sleep laboratories and a control room for supervising researchers will be installed.

Randomization: For logistic reasons, during the study period from July to October, a total number of 52 time slots will be available at the different locations that allow allocating 52 subjects to 4 different sequences of evaluations (sequences A-D, table 1, 13 subjects per sequence) according to a balanced block design. In order to control for a potential order effect, 26 sequences A&C, starting with low altitude studies and ending with high altitude studies, and 26 sequences B&D, starting with high altitude studies and ending with low altitude studies, will be available (figure 1). Sequences A&B and C&D, respectively, vary in the order of exposure to 1631m and 2590m. Details of sequences and the fact that selecting any particular time slot determines the sequence will be concealed to participants until allocation is completed. Randomization will be achieved by letting participants register for one of the available time slots according to their preference (of any date) without being aware of the corresponding sequence until all slots are filled.

7.1.1 **Schedule of daily assessments study phase 1**

Table 3. Schedule of daily assessments study phase 1		
from	to	Activity
	17:00	arrival, transfer to rooms
17:15	18:00	Clinical examination, questionnaires
18:00	19:00	Dinner
19:00	19:45	Driving simulator
19:45	20:30	Psychomotor vigilance test
20:30	21:15	Snowboard-simulator
21:15	22:00	Motor Task Manager
22:00	06:00	Sleep study
06:00	08:00	venous blood sampling, clinical examination, questionnaires
08:00	08:45	Breakfast
08:45	09:30	Driving simulator
09:30	10:15	Psychomotor vigilance test
10:15	11:00	Snowboard-simulator
11:00	11:45	Motor Task Manager
11:45	12:30	Pulse wave analysis
12:30	13:30	Lunch
13:30	14:00	Spirometry&DLCO
14:00	17:00	Leisure time (transfer to next location if applicable)

The time table represents one example of a sequence of evaluations. During the assessment periods in the evenings and mornings subjects will rotate through different sequences of tests each lasting for 45 min. The order of tests will vary according to availability of measurement techniques.

7.2 DETAILED STUDY DESIGN FOR THE STUDY PHASE 2 EVALUATING THE COMBINED EFFECTS OF SLEEP RESTRICTION AND ALTITUDE

The purpose is to investigate the combined effects of hypobaric hypoxia at altitude and sleep restriction on sleep, breathing, psychomotor performance and cardiovascular function. A randomized, 2x2 balanced cross-over design will be used to control for the factors altitude and sleep restriction, respectively. The study will comprise 2 periods of 4 days, i.e. a total of 8 days. Within each study period, 2 days will be spent in Zurich and 2 days at altitude (Davos Jakobshorn, 2590m). The two periods will be separated by a 7 days wash-out period. In the week before study entry and during the wash-out period, subjects will maintain mandatory bed times in darkness from 22:00 to 06:00 h verified by actimetry. During the 4 h sleep restriction, sleep will be scheduled between 02:00 to 06:00h.
Subjects will be informed to refrain from driving during and on the day after the phase of sleep restriction.
During non-restriction trials subjects will have mandatory bed times in darkness between 22:00 to 06:00h. After recruitment, individuals will be randomized to one of 4 different sequences of studies as shown in table 4 and figure 3. Evaluations at all locations will be identical.

Table 4. Sequences of evaluation in study phase 2

Day -60 to 0: Subject screening, recruitment, randomization			
Sequence A	Sequence B	Sequence C	Sequence D
Restricted sleep<br>490m, 2 days<br>2590m, 2 days	*Restricted sleep*<br>2590m, 2 days<br>490m, 2 days	*Normal Sleep*<br>490m, 2 days<br>2590m, 2 days	*Normal Sleep*<br>2590m, 2 days<br>490m, 2 days
7 d wash-out	7 d wash-out	7 d wash-out	7 d wash-out
Normal Sleep<br>490m, 2 days<br>2590m, 2 days	*Normal Sleep*<br>2590m, 2 days<br>490m, 2 days	*Restricted sleep*<br>490m, 2 days<br>2590m, 2 days	*Restricted sleep*<br>2590m, 2 days<br>490m, 2 days

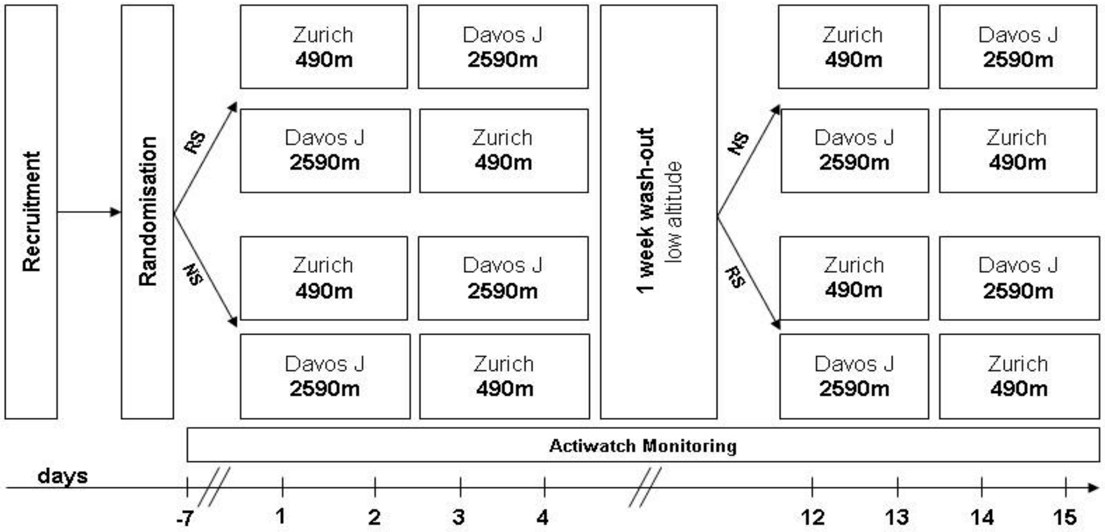

Figure 3: Study phase 2 design representing subject flow and the 4 different sequences of evaluations. Study locations comprise the Clinical Trial Center, University Hospital of Zurich (Zurich, 490m) and the Hotel at Jakobshorn, Davos (Davos J, 2590m). At both locations single room sleep laboratories and a control room for supervising researchers will be installed.

Randomization: This is performed in a similar way as for study phase 1 (see above)

7.2.1 Schedule of daily assessments study phase 2, normal sleep

During normal sleep periods subjects will have mandatory bed-times in darkness between 22:00 to 06:00h. Bed-times and darkness will be verified by the supervising researchers.

Table 5. Daily evaluations study phase 2, *normal* sleep

from	to	Activity
	17:00	arrival, transfer to rooms
17:15	18:00	Clinical examination, questionnaires
18:00	19:00	Dinner
19:00	19:45	Driving simulator
19:45	20:30	Psychomotor vigilance test
20:30	21:15	Snowboard-simulator
21:15	22:00	Motor Task Manager
22:00	06:00	Sleep study
06:00	08:00	venous blood sampling, clinical examination, questionnaires
08:00	08:45	Breakfast
08:45	09:30	Driving simulator
09:30	10:15	Psychomotor vigilance test
10:15	11:00	Snowboard-simulator
11:00	11:45	Motor Task Manager
11:45	12:30	Pulse wave analysis
12:30	13:30	Lunch
13:30	14:00	Spirometry&DLCO
14:00	17:00	Leisure time (transfer to next location if applicable)

7.2.2 Schedule of daily assessments study phase 2, restricted sleep

Restricted sleep means supervised sleep duration of 4 h per night between 02:00 to 06:00h with mandatory bed-times in darkness between 2:00 to 06:00h. Sleep restriction will be ensured by supervised entertainment (e.g. board/card games, watching TV) until bed-time at 02:00.

Table 6. Daily evaluations study phase 2, *restricted* sleep

from	to	activity
	17:00	arrival, transfer to rooms
17:15	18:00	Clinical examination, questionnaires
18:00	19:00	dinner
19:00	19:45	Driving simulator
19:45	20:30	Psychomotor vigilance test
20:30	21:15	Snowboard-simulator
21:15	22:00	Motor Task Manager
22:00	02:00	Evening program to keep subjects awake (see above)
02:00	06:00	Sleep study
06:00	08:00	venous blood sampling, clinical examination, questionnaires
08:00	08:45	Breakfast
08:45	09:30	Driving simulator
09:30	10:15	Psychomotor vigilance test
10:15	11:00	Snowboard-simulator
11:00	11:45	Motor Task Manager
11:45	12:30	Pulse wave analysis
12:30	13:30	lunch
13:30	14:00	Spirometry&DLCO
14:00	17:00	Leisure time (transfer to next location if applicable)

7.3 STUDY OUTCOME MEASURES

7.3.1 Primary outcomes

- Sleep disordered breathing (number of apneas/hypopneas)
- Sleep quality (sleep efficiency, amount of slow wave sleep)
- Vigilance (Psychomotor vigilance test reaction time)
- Cardiovascular function (mean nocturnal heart rate)

7.3.2 Additional outcomes

- Breathing patterns, ventilation and gas exchange assessed by noninvasive means (oxygen saturation, transcutaneous and end-tidal carbon dioxide tension, respiratory inductive plethysmography to measure minute ventilation and spirometry as well as carbon monoxide diffusion)

- Quantitative sleep analysis assessed by EEG power spectral analysis and by slow wave morphometry
- Subjective sleep quality by visual analog scale
- Psychomotor performance and vigilance assessed by the Motor Task Manager, a computer based test of complex motor skills; driving simulator and snow board simulator performance
- ECG derived heart rate, arrhythmia, heart rate variability measures of autonomic function; blood pressure, indices of arterial stiffness and endothelial function assessed by sphygmomanometry and noninvasive applanation tonometry of the radial artery
- Blood markers of systemic inflammation (CRP, IL-6, TNF-alpha, TGF-beta), cardiovascular function (BNP, cell-derived micro particles) and insulin sensitivity.

8 Participant Entry

8.1 Pre-registration evaluations

Medical history and current health issues will be evaluated. Sleepiness will be assessed by means of the Stanford Sleep Disorders Questionnaire[14;15] and the Epworth sleepiness scale[14]. A physical examination including weight, height, blood pressure and cardio-pulmonary auscultation will be performed.

8.2 Inclusion criteria

- Healthy, i.e. absence of any previous or current medical condition other than minor intercurrent medical issues or accidents that do not interfere with current health status or physical performance. No current medical treatment.
- male gender (inclusion of females is not feasible because it would require to control for the effects of the menstrual cycle on ventilation and various other physiological processes).
- age 20-70y
- residence at <800m
- informed consent.

8.3 Exclusion criteria

- Any active disease or health related condition requiring treatment (in particular, sleep apnea and other sleep disorders, chronic rhinitis, cardiovascular and lung disease)
- body mass index <18 or >30 kg/m^2
- use of drugs that affect respiratory center drive (benzodiazepines or other sedatives, sleep inducing drugs, morphine or codeine derivates), stimulants (modafinil, methylphenidate, theophylline), or illicit drugs
- Previous intolerance of moderate altitude (<3000m) due to acute mountain sickness, altitude related cerebral or pulmonary edema requiring unplanned descent, medical treatment or evacuation
- Exposure to altitudes >1500m for >1 day within the last 2 weeks before the study
- salbutamol-/nitroglycerin-intolerance
- Regular consumption of recreational drugs including alcohol and nicotine

8.4 Withdrawal criteria

Any active or unstable disease leads to exclusion from our study. Consumption of alcohol, illicit drugs, medication as described above or any unruly behavior or failure to follow the study protocol leads also to exclusion. Participants can withdraw from the study anytime without reason.

9 Efficacy and Safety Variables

9.1 EFFICACY VARIABLES

9.1.1 Symptom evaluation

Subjective sleep quality will be evaluated by a visual analog scale. Subjective sleepiness will be evaluated by the Stanford Sleep Disorder Questionnaire. Symptoms of acute mountain sickness will be evaluated by the Environmental Symptoms Questionnaire cerebral score (AMS-c)[16].

9.1.2 Sleep and breathing

9.1.2.1 Polysomnography

Nocturnal polysomnography are performed according to standard techniques as described previously [17]. In addition to central EEG derivations, EOG and submental EMG, measurements include calibrated respiratory inductive plethysmography (RespiratracePT, Nims, Miami Beach, USA), pulse oximetry, end-tidal [18] (CapnoCheck Plus, ResMed, Basel Switzerland) and transcutaneous PCO_2 [19] (Tosca, Radiometer, Basel, Switzerland), pulse transit time [20], nasal airflow estimated by nasal prongs connected to a pressure transducer [21], oral thermistor, bilateral sub costal surface EMG of the diaphragm [22] to assist in differentiation of obstructive from central apnea/hypopnea. Sleep and arousals will be scored according to standard criteria as described by Rechtschaffen und Kales and by the American Academy of Sleep Medicine guidelines [23;24]. An apnea or hypopnea will be scored if the amplitude of the nasal pressure swings or the sum signal of the respiratory inductive plethysmography falls below 50% of baseline during the previous 2 minutes for >10 seconds [25].

9.1.2.2 Pulmonary function testing

In order to detect subclinical High Altitude Pulmonary Edema Spirometry including diffusion capacity will be performed according to standard techniques[A3, A4]. In cases of subclinical HAPE vital capacity as well as the diffusion capacity will be reduced due to increased intrapulmonary fluid accumulation.

A3) ATS/ERS task force. Standardization of spirometry. Eur Respir J 2005;26:319-338.
A4) ATS/ERS task force. Standardization of the single breath uptake of carbon monoxide in the lung. Eur Respir J 2005;26:720-735. A2) O. Senn et al.. Med Sci.Sports Exerc. 38 (9):1565-1570, 2006.

9.1.3 Vigilance and psychomotor performance

These tests as described below provide multiple, well defined parameters of psychomotor performance that are sensitive to sleep[26]. In addition, driving[27;28] and snow board[29] simulators are used to evaluate complex and realistic skill performance. All tests will be performed in a quiet room with subjects wearing sound protective ear covers. The light in the room will be dimmed.

9.1.3.1 Psychomotor Vigilance Test (PVT)

Vigilance will be assessed by the Psychomotor Vigilance Test (PVT) which is sensitive for effects of sleep restriction/disturbance and hypoxia [7;30]. As described by Dinges et al[31] the PVT measures the reaction time to a visual stimulus (LED) presented in variable intervals between 1 to 10 s over a period of 10-15 min. The subjects respond by pressing a button on a hand-held device (Stowood Scientific Instruments Ltd., Oxford).

9.1.3.2 Motor Task Manager (MTM)

The MTM[32] requires the subjects to move a cursor with a hand on the digitizing surface while their hand position and target locations are displayed on a computer screen. A computer program reports the main extracted parameters such as performed trajectories; speed, acceleration and jerk profiles, directional error, linear error and time error. (ETT s.r.l., Genova)

9.1.3.3 Divided Attention Driving Simulator (DADS)

The DADS[27;28] is a personal computer based driving simulation test. While driving down the abstract image of a winding road, random single digit numbers will be displayed at the screen corners. Subjects are required to confirm the appearance of the number ‘2’ in addition to keeping the virtual car on track. (SimDrive, Divided Attention Driving Simulator, Stowood Scientific Instruments, Oxford, UK). Outcomes are mean distance from center (mean error), SD of mean distance from center, number of off-road events, reaction time (time to response to digit 2).

9.1.3.4 Snowboarding Simulation

A commercial entertainment system developed by Nintendo Co is being used to test the motor performance by means of manufacturer provided and custom software with the snowboarding simulation game. With the balance board (a platform equipped with 4 gravity sensors upon which the subjects stand) this system provides a reliable, scientifically evaluated assessment for balance. The Nintendo System compares favorably to ‘gold standard’ laboratory grade force-platforms[29] (Nintendo Wii fit plus with balance board, Nintendo Co., Ltd, Kyoto, Japan).

9.1.4 Cardiovascular function

To assess autonomic function, measures of heart rate variability (SD, low/high frequency spectral power) [33;34] derived by ECG during polysomnography will be analyzed.

9.1.4.1 Augmentation index (AIx) measured by pulse wave analysis

Pulse wave analysis is a technique which uses applanation tonometry to assess the arterial pressure waveform. The pressure within an artery is a composite of both the forward ejected wave and the reflected waves travelling back to the heart. In healthy arteries, the reflected waves travel slowly and reach the heart in diastole thereby enhancing coronary perfusion. If the vasculature is stiffened, the reflected waves travel faster and reach the heart in systole, thus

augmenting systolic pressure[35]. Systemic arterial stiffness can be measured using augmentation index (AIx), calculated as the difference between the second systolic peak and inflection point, expressed as a percentage of the central pulse pressure[35]. AIx decreases in response to vasodilators (e.g. nitro-glycerine, salbutamol) by reduction of smooth muscle tone, and it has been demonstrated that AIx falls following inhaled salbutamol as a result of endothelial dependent nitric oxide release[36]. Therefore, an impaired decrease of AIx after inhalation of salbutamol is a display of endothelial dysfunction. Pulse wave analysis is performed with a micro manometer (SphygmoCor, At-Cor Medical) which records radial artery pulse waveforms continuously. Mean values of approximately 10 pulse waves are used for analyses. A validated transfer function[37] is used to generate a corresponding central aortic pressure waveform as described previously[38] and AIx is then calculated. After a baseline recording, salbutamol is administered by inhalation (400 µg), and a re-evaluation of the pulse wave is performed after 10 minutes. Endothelial independent vasodilatation is assessed by pulse wave analysis as described above 3 minutes after sublingual application of nitro-glycerine 0.5 mg.

9.1.4.2 Blood Markers of inflammation, cardiovascular function and insulin sensitivity

Blood will be drawn in the mornings after each sleep study, centrifuged and resulting plasma samples will be stored at -80 °C for later analysis. The Dade Behring BN method (particle-enhanced immunonephelometry, detection limit 0.18 mg/L, measuring range 0.18-1150 mg/L) will be used to measure HsCRP as previously described and validated[39]. IL-6, TNF-α (inflammatory cytokines) and TGF-β (anti-inflammatory cytokine) will measured by highly sensitive ELISA with commercially available kits (Bender MedSystems Gmbh, Vienna, Austria) with a lower limit of detection of 0.1 pg/ml. Brain natriuretic peptide (BNP), fasting insulin and glucose as well as cell-derived micro particles will be measured as previously described[40;41].

9.2 Safety variables

Adverse events or reactions will be evaluated by daily physical examinations, evaluation of questionnaires, through the acquisition of the variables described above and through voluntary report.

10 Adverse events

10.1 DEFINITIONS

10.1.1 Adverse Event (AE):

Any untoward medical occurrence in a study participant, whether or not related to the study, will be recorded. Each participant will be examined according to checklists and with questionnaires during the study. Adverse events will be recorded in prepared forms and checklists (see CRF).

10.1.2 Serious Adverse Event (SAE):

Any untoward and unexpected medical occurrence or effect that:

- Results in death,
- Is life-threatening – refers to an event in which the subject was at risk of death at the time of the event; it does not refer to an event which hypothetically might have caused death if it were more severe,
- Requires hospitalisation, or prolongation of existing inpatients' hospitalisation,
- Results in persistent or significant disability or incapacity,
- Is a congenital anomaly or birth defect.
- Medical judgement should be exercised in deciding whether an AE is serious in other situations. Important AEs that are not immediately life-threatening or do not result in death or hospitalisation but may jeopardise the subject or may require intervention to prevent one of the other outcomes listed in the definition above, should also be considered serious.

SAEs will be immediately reported to the Ethics committee of the Canton Zürich. A special form will be completed. Patients will be followed until the event resolves or until 12 weeks after the event is reported.

An emergency action plan will be prepared with instructions on first aid and evacuation. In case of an emergency at Davos Jakobshorn the patient will receive first aid and treatment and will be evacuated to the hospital of Davos if necessary. Descent by cable car is available for emergencies also at nighttimes. First aid materials will be readily available at all study locations.

Since this is a physiologic study in healthy subjects no serious or clinically relevant adverse effects are expected. Light forms of Acute Mountain Sickness with symptoms such as headache, light-headedness, fatigue and insomnia may occur. More severe symptoms are unlikely because of the moderate altitude. Nevertheless, oxygen administration to reverse hypoxia and subsequent descent to lower altitude may be performed in case of emergencies. Skin irritation may appear due to attachments of the sensors. Venous blood will be drawn by a physician. Minor pain and hematoma at the puncture site may occur. A first aid kit with medication for treatment of altitude related illness and for skin irritation and hematoma from venipuncture will be readily available.

10.2 REPORTING PROCEDURES

All adverse events will be reported. Depending on the nature of the event the reporting procedures below should be followed. Any questions concerning adverse event reporting will be directed to the Principal Investigator in the first instance.

10.2.1 Non serious AEs

All such events, whether expected or not, will be recorded.

10.2.2 Serious AEs

A SAE form will be completed and forwarded to the Principal Investigator within 24 hours.

The Principal Investigator will assess whether the event is:

- ‘related’, i.e. resulted from the administration of any of the research procedures; and
- ‘unexpected’, i.e. an event that is not listed in the protocol as an expected occurrence.

Reports of suspected related and unexpected SAEs (SUSARs) should be submitted within 15 days and within 7 days for fatal or life-threatening SUSARs, respectively, of the investigator becoming aware of the event, to the Ethics Committee using the CIOMS format.

11 ASSESSMENT AND FOLLOW-UP

11.1 RECRUITMENT

Recruitment will include assessment of in- and exclusion criteria, signing the inform consent, assessment of personal history and a clinical examination.

11.2 DAY 1-5 (PHASE 1) AND DAY 1-8 (PHASE 2)

Baseline evaluation in Zürich as well as the daily evaluation in the other study locations (Davos Wolfgang and Jakobshorn) will consist of a clinical examination, questionnaires, polysomnography, vigilance and psychomotor performance tests and cardiovascular assessments as described above. In study phase 1, there is a defined end of the study after the 5th night of examination and morning physical examination as described above. In study phase 2, there is a defined end of the study after the 8th night of examination and morning physical examination as described above.

11.3 FOLLOW-UP

There is no follow-up.

12 Statistics and data analysis

Completeness, plausibility and distribution of data will be assessed by inspection of numerical results and graphical display. Normality of distribution will be tested by the Kolmogorov-Smirnov statistic [42]. Data will be summarized by means (SD) and medians (quartiles) for normal and non-normal distributions. Effects of treatment and altitude will be evaluated by ANOVA and by multiple regression analysis.

Clinically relevant differences in primary outcomes and corresponding SD are defined as follows according to previous studies [7,40, 51,52]: Apnea/hypopnea index: difference 15/h (SD 25/h); prevalence of slow wave sleep relevant difference: 10% of total sleep time (SD 10%); mean reaction time 25ms (SD 30ms)[31;43]; heart rate 10 beats/min (SD 10). Based on these assumptions, power calculation indicates a required sample size of 50 patients to detect clinically relevant differences in primary outcomes with a power of 80% and alpha =0.05 (incorporating a Bonferroni correction to account for multiple comparisons). A total of 56 subjects will be recruited to account for potential drop-outs.
Data and all appropriate documentation will be stored for a minimum of 10 years after the completion of the study.

Before analyzing, all data are anonymized by allocating a numerical code to each participant. The code is stored at the principal investigator's office.

13 Regulatory issues

13.1 ETHICS APPROVAL

The Principal Investigator has obtained approval from the Ethics Committee. The Principal Investigator will require a copy of the Ethic's approval letter before accepting participants into the study. The study will be conducted in accordance with principles enunciated in the current Declaration of Helsinki, the guidelines of Good Clinical Practice (GCP) issued by ICH, and Swiss regulatory authority's requirements.

13.2 CONSENT

Consent to enter the study must be sought from each participant only after a full explanation has been given, an information leaflet offered and time allowed for consideration. Signed participant consent should be obtained. The right of the participant to refuse to participate without giving reasons must be respected.

After the participant has entered the study the clinician remains free to give alternative treatment to that specified in the protocol at any stage if he/she feels it is in the participant's best interest, but the reasons for doing so should be recorded. In these cases the participants remain within the study for the purposes of follow-up and data analysis. All participants are free to withdraw at any time from the protocol treatment without giving reasons and without prejudicing further treatment.

13.3 CONFIDENTIALITY

The Principal Investigator will preserve the confidentiality of participants taking part in the study and is registered under the Data Protection Act.

13.4 INSURANCE

Insurance is covered by "Haftpflichtversicherung für den Kanton Zürich betreffend das UniversitätsSpital Zürich" (Policy no 9.730.682).

So as not to forfeit their insurance cover, the subjects themselves must comply with the following conditions: Any deterioration in the subject's state of health that may have occurred as a result of the clinical trial must be reported immediately to the investigator, so he or she can notify the insurance company. The subject and his or her parents must take any appropriate measures that may help to determine the cause or the extent of damage, and to minimize the damage. In the event of a subject's death, the insurer must be notified immediately. The subject must not be involved in any other clinical trial during the course of this trial, nor within a period of 30 days prior to its beginning or 30 days after its completion.

13.5 FUNDING

The Zurich Center for Integrative Human Physiology and the Zurich University Hospital are funding this study.

13.6 AUDITS

The study may be subject to inspection and audit by regulatory bodies to ensure adherence to GCP, national law, and regulatory requirements.

14 Study Management

The day-to-day management of the study will be coordinated through Prof. Dr. K. Bloch, Dr. med. T. Latshang, Dr. med. C. Lo Cascio.

14.1 DATA MANAGEMENT

The trial master file will be documented and stored in electronic and paper form at the Klinik für Pneumologie. Zurich University Hospital in room C-RAE 34, and is therefore accessible to authorized medical and research staff only.

15 Publication Policy

The study's results will be presented as scientific papers in medical or physiologic publications.

16 Reference List

(1) Naughton MT, Lorenzi-Filho G. Sleep in heart failure. *Prog Cardiovasc Dis* 2009; 51(4):339-349.

(2) Nussbaumer-Ochsner Y. Lessons from high altitude physiology. Bloch KE, editor. Breath 4[2], 122-132. 1-12-2007. European Respiratory Society.

(3) Pack AI. Advances in sleep-disordered breathing. *Am J Respir Crit Care Med* 2006; 173(1):7-15.

(4) Lanfranchi PA, Colombo R, Cremona G, Baderna P, Spagnolatti L, Mazzuero G et al. Autonomic cardiovascular regulation in subjects with acute mountain sickness. *Am J Physiol Heart Circ Physiol* 2005; 289(6):H2364-H2372.

(5) Baumgartner RW, Eichenberger U, Bartsch P. Postural ataxia at high altitude is not related to mild to moderate acute mountain sickness. *Eur J Appl Physiol* 2002; 86(4):322-326.

(6) van DE, Los M, Dirven P, Sarton E, Valk P, Teppema L et al. Inspired carbon dioxide during hypoxia: effects on task performance and cerebral oxygen saturation. *Aviat Space Environ Med* 2007; 78(7):666-672.

(7) Dinges DF, Pack F, Williams K, Gillen KA, Powell JW, Ott GE et al. Cumulative sleepiness, mood disturbance, and psychomotor vigilance performance decrements during a week of sleep restricted to 4-5 hours per night. *Sleep* 1997; 20(4):267-277.

(8) Pack AI, Maislin G, Staley B, Pack FM, Rogers WC, George CF et al. Impaired performance in commercial drivers: role of sleep apnea and short sleep duration. *Am J Respir Crit Care Med* 2006; 174(4):446-454.

(9) Nussbaumer-Ochsner Y. Breathing disturbances in obstructive sleep apnea patients at high altitude. Schupfer N, lrich S, editors. Am.J.Respir.Crit Care Med. A936. 2008.

(10) Nussbaumer-Ochsner Y. Sleep at altitude. High altitude: human adaptation to hypoxia. New York: Informa Helathcare USA, Inc., 2009. 1-1-2009. New York: Informa Helathcare USA, Inc., 2009.

(11) Steriade M, McCormick DA, Sejnowski TJ. Thalamocortical oscillations in the sleeping and aroused brain. *Science* 1993; 262(5134):679-685.

(12) Sejnowski TJ, Destexhe A. Why do we sleep? *Brain Res* 2000; 886(1-2):208-223.

(13) Tononi G, Cirelli C. Sleep function and synaptic homeostasis. *Sleep Med Rev* 2006; 10(1):49-62.

(14) Bloch KE, Schoch OD, Zhang JN, Russi EW. German version of the Epworth Sleepiness Scale. *Respiration* 1999; 66(5):440-447.

(15) Douglass AB, Bornstein R, Nino-Murcia G, Keenan S, Miles L, Zarcone VP, Jr. et al. The Sleep Disorders Questionnaire. I: Creation and multivariate structure of SDQ. *Sleep* 1994; 17(2):160-167.

(16) Sampson JB, Cymerman A, Burse RL, Maher JT, Rock PB. Procedures for the measurement of acute mountain sickness. *Aviat Space Environ Med* 1983; 54(12 Pt 1):1063-1073.

(17) Bloch KE. Polysomnography: a systematic review. *Technol Health Care* 1997; 5(4):285-305.

(18) Kohler M, Kriemler S, Wilhelm EM, Brunner-LaRocca H, Zehnder M, Bloch KE. Children at high altitude have less nocturnal periodic breathing than adults. *Eur Respir J* 2008; 32(1):189-197.

(19) Senn O, Clarenbach CF, Kaplan V, Maggiorini M, Bloch KE. Monitoring carbon dioxide tension and arterial oxygen saturation by a single earlobe sensor in patients with critical illness or sleep apnea. *Chest* 2005; 128(3):1291-1296.

(20) Pitson DJ, Sandell A, van den Hout R, Stradling JR. Use of pulse transit time as a measure of inspiratory effort in patients with obstructive sleep apnoea. *Eur Respir J* 1995; 8(10):1669-1674.

(21) Thurnheer R, Xie X, Bloch KE. Accuracy of nasal cannula pressure recordings for assessment of ventilation during sleep. *Am J Respir Crit Care Med* 2001; 164(10 Pt 1):1914-1919.

(22) Maarsingh EJ, van Eykern LA, Sprikkelman AB, Hoekstra MO, van Aalderen WM. Respiratory muscle activity measured with a noninvasive EMG technique: technical aspects and reproducibility. *J Appl Physiol* 2000; 88(6):1955-1961.

(23) EEG arousals: scoring rules and examples: a preliminary report from the Sleep Disorders Atlas Task Force of the American Sleep Disorders Association. *Sleep* 1992; 15(2):173-184.

(24) Rechtschaffen A. *A manual of standardized terminology, techniques and scoring system for sleep stages of human subjects*. Kales A, editor. 2010. Washington, D.C.: Public Health Service, U.S. Government Printing Office.

(25) Sleep-related breathing disorders in adults: recommendations for syndrome definition and measurement techniques in clinical research. The Report of an American Academy of Sleep Medicine Task Force. *Sleep* 1999; 22(5):667-689.

(26) Huber R, Ghilardi MF, Massimini M, Tononi G. Local sleep and learning. *Nature* 2004; 430(6995):78-81.

(27) Juniper M, Hack MA, George CF, Davies RJO, Stradling JR. Steering simulation performance in patients with obstructive sleep apnoea and matched control subjects. *European Respiratory Journal* 2000; 15(3):590-595.

(28) Mazza S, Pepin JL, Naegele B, Rauch E, Deschaux C, Ficheux P et al. Driving ability in sleep apnoea patients before and after CPAP treatment: evaluation on a road safety platform. *European Respiratory Journal* 2006; 28(5):1020-1028.

(29) Clark RA, Bryant AL, Pua Y, McCrory P, Bennell K, Hunt M. Validity and reliability of the Nintendo Wii Balance Board for assessment of standing balance. *Gait Posture* 2009.

(30) Gerard AB, McElroy MK, Taylor MJ, Grant I, Powell FL, Holverda S et al. Six percent oxygen enrichment of room air at simulated 5,000 m altitude improves neuropsychological function. *High Alt Med Biol* 2000; 1(1):51-61.

(31) Dinges DF, Powell JW. Microcomputer Analyses of Performance on A Portable, Simple Visual Rt Task During Sustained Operations. *Behavior Research Methods Instruments & Computers* 1985; 17(6):652-655.

(32) Warner MB. Motor task manager and upper limb reaching task: Validation of movement analysis . Novellino A, editor. Gait & Posture 30, 57. 2010. Elsevier B.V.

(33) Heart rate variability: standards of measurement, physiological interpretation and clinical use. Task Force of the European Society of Cardiology and the North American Society of Pacing and Electrophysiology. *Circulation* 1996; 93(5):1043-1065.

(34) Wilkinson IB, Fuchs SA, Jansen IM, Spratt JC, Murray GD, Cockcroft JR et al. Reproducibility of pulse wave velocity and augmentation index measured by pulse wave analysis. *J Hypertens* 1998; 16(12 Pt 2):2079-2084.

(35) Hayward CS, Kraidly M, Webb CM, Collins P. Assessment of endothelial function using peripheral waveform analysis: a clinical application. *J Am Coll Cardiol* 2002; 40(3):521-528.

(36) Dawes M, Chowienczyk PJ, Ritter JM. Effects of inhibition of the L-arginine/nitric oxide pathway on vasodilation caused by beta-adrenergic agonists in human forearm. *Circulation* 1997; 95(9):2293-2297.

(37) Pauca AL, O'Rourke MF, Kon ND. Prospective evaluation of a method for estimating ascending aortic pressure from the radial artery pressure waveform. *Hypertension* 2001; 38(4):932-937.

(38) Wilkinson IB, Fuchs SA, Jansen IM, Spratt JC, Murray GD, Cockcroft JR et al. Reproducibility of pulse wave velocity and augmentation index measured by pulse wave analysis. *J Hypertens* 1998; 16(12 Pt 2):2079-2084.

(39) Roberts WL, Moulton L, Law TC, Farrow G, Cooper-Anderson M, Savory J et al. Evaluation of nine automated high-sensitivity C-reactive protein methods: implications for clinical and epidemiological applications. Part 2. *Clin Chem* 2001; 47(3):418-425.

(40) Ayers L, Ferry B, Craig S, Nicoll D, Stradling JR, Kohler M. Circulating cell-derived microparticles in patients with minimally symptomatic obstructive sleep apnoea. *Eur Respir J* 2009; 33(3):574-580.

(41) Kohler M, Ayers L, Pepperell JC, Packwood KL, Ferry B, Crosthwaite N et al. Effects of continuous positive airway pressure on systemic inflammation in patients with moderate to severe obstructive sleep apnoea: a randomised controlled trial. *Thorax* 2009; 64(1):67-73.

(42) Kirkwood BR. Essential medical statistics. 2003.

(43) Muhm JM, Signal TL, Rock PB, Jones SP, O'Keeffe KM, Weaver MR et al. Sleep at simulated 2438 m: effects on oxygenation, sleep quality, and postsleep performance. *Aviat Space Environ Med* 2009; 80(8):691-697.

17 Appendices

- Patient Information Sheet/ Informed Consent Form
- Case Report Form (CRF)
- Public study advertisement
- Study protocol summary in German
- Electronic Version of the protocol and further documentation on CD-Rom

18 Declarations of Sponsor-Investigator and Investigator

18.1 DECLARATION OF SPONSOR-INVESTIGATOR

This clinical trial protocol was subject to critical review and has been approved by the Sponsor-Investigator. The information herein is consistent with

- the current risk/benefit evaluation of the investigational procedure(s)
- the moral, ethical and scientific principles governing clinical research as set out in the Declaration of Helsinki, Good Clinical Practice *and SAMW* guidelines.

Sponsor-Investigator

Zurich, 04.05.2010 ______________________ ______________________

Konrad E. Bloch

18.2 DECLARATION OF INVESTIGATOR

I have read this trial protocol and aggress to conduct the trial as set out in this protocol.

Investigators

Ts. Latshang

Zurich, 04.05.2010 ______________________

Tsogyal Latshang

Zurich, 04.05.2010 ______________________

Christian Lo Cascio

Zurich, 04.05.2010 K. Stadelmann

Katrin Stadelmann

Zurich, 04.05.2010 ______________________

Malcolm Kohler

Zurich, 04.05.2010 P. Achermann

Peter Achermann

Zurich, 04.05.2010 ______________________

Reto Huber
